# Uncovering the spatiotemporal evolution of the service industry based on geo-big-data- a case study on the bath industry in China

**DOI:** 10.1007/s43762-023-00085-y

**Published:** 2023-02-27

**Authors:** Bingyu Zhao, Jingzhong Li, Bing Xue

**Affiliations:** 1 Institute of Applied Ecology, Chinese Academy of Sciences, No.72 Wenhua Road, Shenyang, 110016 China; 2 College of Urban Planning and Architecture, Xuchang University, No. 88 Bayi Road, Xuchang, 461000 China

**Keywords:** Bath industry, Spatial pattern, Points of interest data, Population migration data, Radial basis function neural network, China

## Abstract

The bath industry has multiple attributes, such as economic, health, and cultural communication. Therefore, exploring this industry's spatial pattern evolution is crucial to forming a healthy and balanced development model. Based on POI (Points of Interest) and population migration data, this paper uses spatial statistics and radial basis function neural network to explore the spatial pattern evolution and influencing factors of the bath industry in mainland China. The results show that: (1) The bath industry presents a strong development pattern in the north, south-northeast, and east-northwest regions and weak development in the rest of the country. As a result, the spatial development of new bath space is more malleable. (2) The input of bathing culture has a guiding role in developing the bath industry. The growth of market demand and related industries has a specific influence on the development of the bath industry. (3) Improving the bath industry's adaptability, integration, and service level are feasible to ensure healthy and balanced development. (4) Bathhouses should improve their service system and risk management control during the pandemic.

## Introduction

The bath industry has multiple attributes, such as economic, health (Zhao et al., 2021), and cultural communication (Xue et al., 2023a, 2023b), which serve as an essential industry to improve people's life quality and stimulate socio-economic development (Ibem and Amole 2013; Xue et al., 2018). In addition, the 2030 United Nations Sustainable Development Goal (SDG) proposes to 'ensure a healthy lifestyle and promote the well-being of people of all ages' (United Nations, 2015). Various countries and regions have also formulated specific policies to ensure the development of related industries to promote the realization of SDG goals according to national conditions. For example, Japan proposed health tourism based Hot Spring to promote the development of the industry(Yoichi, 2020). The Chinese Ministry of Commerce and other departments 2021 propose to enhance bathing services in rural marketplaces, thus improving the quality of rural living services (Ministry of Commerce of the People's Republic of China, 2021).

Earlier studies about the bath industry focused on cultural perspectives, mainly on the causes of bath culture, related allusions, and bath characteristics (Vollrath, 1999). For example, Bodolai and Kósa (2018) introduced and constructed a conceptual map of the Memi Pasha bath in Turkish bath culture; Koo and Jun (2012) argued that bathing is a positive method for treating diseases in Korean society, and the time and climate of bathing may lead to different healing effects; Han (2019) argued that Chinese bath culture is driven by urbanization and economic prosperity. There are ancient-modern and north–south differences in the Chinese bathing culture; however, with the optimization and upgrading of economic culture (Yao et al., 2021), the bath industry is forming toward an industrial scale, such as the Japanese spa tourism industry (Kurata and Ohe 2020). As a new economic-geographical phenomenon, the bath industry's spatial development pattern, process, and influencing factors have attracted the attention of geographers (Guan et al., 2018). For example, Kamata and Misui (2015) studied the different motivations of Japanese tourists spending time in hot springs on weekends and weekdays through questionnaires; Jiang (2018) found that there was a spatial mismatch between the supply and demand of recreational and health care resources such as bath industry in Nanjing. The above studies have played a positive role in a better understanding of the bath industry (Zhu and Zhang 2021). However, a systematic understanding of the spatial development of the bath industry at the national level has not been observed yet; therefore, it failed to provide decision support for the healthy and sustainable development of the bath industry in the global context (Bonakdar & Audirac, 2021; Follmann et al., 2021).

Today, applying new geo-big data offers a new way to uncover the spatial patterns of industry development. For example, the POI data which includes various information like feature name, type, latitude, and longitude, showing the characteristics of large volume, accuracy, and timeliness (Xue et al., 2019), providing the possibility to precisely depict the vitality and spatial development of economic sectors (Fan & Stewart, 2021; Liu & Yao, 2021). For example, Xue et al., (2020a, 2020b) used POI data to find that the equipment manufacturing industry and automobile sales industry in Shenyang show a spatial complementary and integrated relationship; Wu et al., (2021a, 2021b) used POI, subway location, and other multi-source data to classify the factors affecting the rise and fall of restaurants in Beijing into four aspects: promotion, hindrance, stability, and irrelevance; Zhu (2021) identified the spatial diffusion from local food to national snacks with the help of POI data.

In 2010, the Ministry of Commerce of the People's Republic of China issued the 'Guidance of the Ministry of Commerce on the Standardization and Development of the Bath industry' (2010), pointing out the need to optimize the bath industry structure based on local economic development and people's needs. Furthermore, in 2016, the State Council of the People's Republic of China issued the 'Outline of the Healthy China 2030 Plan' (2016), suggesting promoting the integration of health, bath, and leisure industries and the development of a new business health services model. As a result, the developmental scale of China's bath industry has been expanding, with turnover reaching 345.62 billion yuan in 2016 (Ministry of Commerce of the People's Republic of China, 2017), but the spatial development pattern of the industry and the influencing factors remain ambiguous (Calafiore et al. 2021; You et al., 2021), which hinder the comprehensive assessment of the implementation effects of relevant policies in China and the contribution and gap for the implementation of the SDGs goals (Jiao et al., 2021; Li 2019). Therefore, based on multiple sources of data such as POI, population migration and statistical data, this paper attempts to discover the spatial pattern and influencing factors of China's bathing industry, providing case support and methodological reference for research on the spatial development of local special industries, and providing support for promoting the healthy and sustainable development of the bathing industry.

## Data sources and methodology

### Data acquisition and pre-processing

The data sources in this paper mainly include 1) 2008 and 2018 POI data obtained through the AMAP API interface; after pre-processing such as coordinate conversion, cleaning, and cross-checking, the required data is obtained regarding 'AMAP POI Classification Code' (https://lbs.amap.com/api/webservice/download/). In Table 1, 'bath industry' is a service form based on comprehensive baths, foot baths, SPA, and recreation; 'traditional bath' refers to providing daily cleaning services for residents, which does not have recreational and health effects; 'new bath' includes a variety of functions such as clean body, health, recreational entertainment; 'catering services' refers to industries that operate or provide catering; 'accommodation services' refers to industries that provide customers with accommodation, catering and a variety of integrated services; 'shopping services' refers to services that revolve around daily matters such as clothing, food, housing, transportation, shopping and entertainment; 'entertainment establishments' refers to places that are open to the public for profit and for consumers to entertain themselves. 2) Population migration data is Tencent location data (https://heat.qq.com/bigdata/index.html) for the 2018 Spring Festival return peak period (February 20, 2018-February 26, 2018); 3) The Statistics are from *the 2009 China City Statistical Yearbook*, *2019 China City Statistical Yearbook,* and *China Economic and Social Big Data Research Platform* (https://data.cnki.net/). 4) The administrative boundary data include the administrative boundaries of prefecture-level cities and the boundaries of four major geographic regions in China, which were obtained from *the Resource and Environment Science and Data Center of the Chinese Academy of Sciences* (https://www.resdc.cn/). The four geographic regions include the northern, southern, northwestern, and Qinghai-Tibet regions, and the smallest research units are municipalities directly under the central government, sub-provincial cities, prefecture-level cities, autonomous regions, and county-level administrative regions under provincial administration in mainland China, with a total of 364 geographic units.

**Table 1:** Main categories of POI data

	Year	Quantity
Bath industry	2008; 2018	20,062; 63,053
Traditional bath	2008; 2018	6472; 15,578
New bath	2008; 2018	13,590; 47,475
Catering Industry	2018	3,544,778
Accommodation industry	2018	804,922
Shopping Industry	2018	6,178,554
Entertainment Industry	2018	384,608

### Methodology

#### Mathematical statistics

Mathematical statistics is a powerful tool to describe the regular quantitative characteristics of geographic elements (Milias & Psyllidis, 2021) and identify the development and changing data patterns in space. In this paper, we calculate the total number of POIs and the growth rate at the scale of four major geographic areas and municipalities, respectively, and reveal the spatial variability of density, growth amount, and growth rate of the bath industry through visualization (Martin et al., 2022).

#### Spatial autocorrelation

Global spatial autocorrelation (Moran's I) is an effective technique to measure the spatial density variation and spatial clustering effect of elements (Shaikh et al., 2021). It establishes a spatial weight matrix to measure the spatial autocorrelation and variation of elemental attribute values, reflecting the clustering and correlation of elements in the region. The spatial autocorrelation is divided into two scales of global and local measures, and the global Moran's I index and local Moran's I are used to test the spatial dependence of the bath industry and its sub-categories in mainland China on the overall and local geography, calculated as

1 $$Mora{n}^{^{\prime}}s\:I=\frac{n\sum_{i=1}^{n}\sum_{j=1}^{n}{w}_{ij}\left({x}_{i}-\overline{x }\right)\left({x}_{j}-\overline{x }\right)}{{S}^{2}\sum_{i=1}^{n}\sum_{j=1}^{n}{w}_{ij}}$$

The global spatial autocorrelation cannot effectively express the spatial autocorrelation between adjacent research units within the region, so Local Indicators of Spatial Association (LISA) are added to form a supplementary explanation of the LISA map (Builes-Jaramillo 2018). Local Moran's *I* is often used, and the formula is:

2 $${Local\:Mora{n}^{^{\prime}}s\:I}_{i}=\frac{({x}_{i}-\overline{x })\sum_{j=1}^{n}{W}_{ij}({x}_{i}-\overline{x })}{{\mathrm{S}}^{2}}$$

where $n$ is the number of spatial units in the study area, and in this paper $n=364$. ${x}_{i}$ and ${x}_{j}$ are the values of adjacent paired spatial units, and $\overline{x }$ denotes the mean value of the attributes in the study area, and ${S}^{2}$ denotes the variance of the bath industry and its sub-categories, and ${w}_{ij}$ is the spatial connectivity matrix between $i$ and $j$. $Mora{n}^{^{\prime}}s I$ denotes the degree of spatial autocorrelation and takes values in the interval [-1,1].$I>0$ indicates positive spatial autocorrelation; $I<0$ indicates negative spatial autocorrelation; $I=0$ indicates no correlation in space.

#### Radial Basis Function (REF) neural network

REF neural network is a multilayer, fully connected feedforward network, including input, implicit, and output layers (Zhang et al., 2020). The working principle is that the signal is transformed nonlinearly from the input layer to the implicit layer by normalizing the radial basis function and then outputted from the implicit layer to the output layer by a linear weighting combination. In this paper, the effect of each influence factor on the spatial clustering and divergence of the bath industry and its subclasses is examined by REF neural network model.

3 $${R}_{i}\left(x\right)={e}^{-\frac{{||{x}_{i}-{{x}_{i}}^{\mathrm{^{\prime}}}||}^{2}}{2{{\sigma }_{i}}^{2}}}$$

where ${R}_{i}\left(x\right)$ denotes the output of the $i$ th node in the hidden layer. ${x}_{i}$ is the n-dimensional input variable, ${x}_{i}$ is the centroid of the Gaussian function of the ith hidden node, and ${\sigma }_{i}$ denotes the normalization parameter of the ith hidden node.

## Results

### Bath industry spatial pattern

#### Spatial heterogeneity pattern

**Fig. 1:**
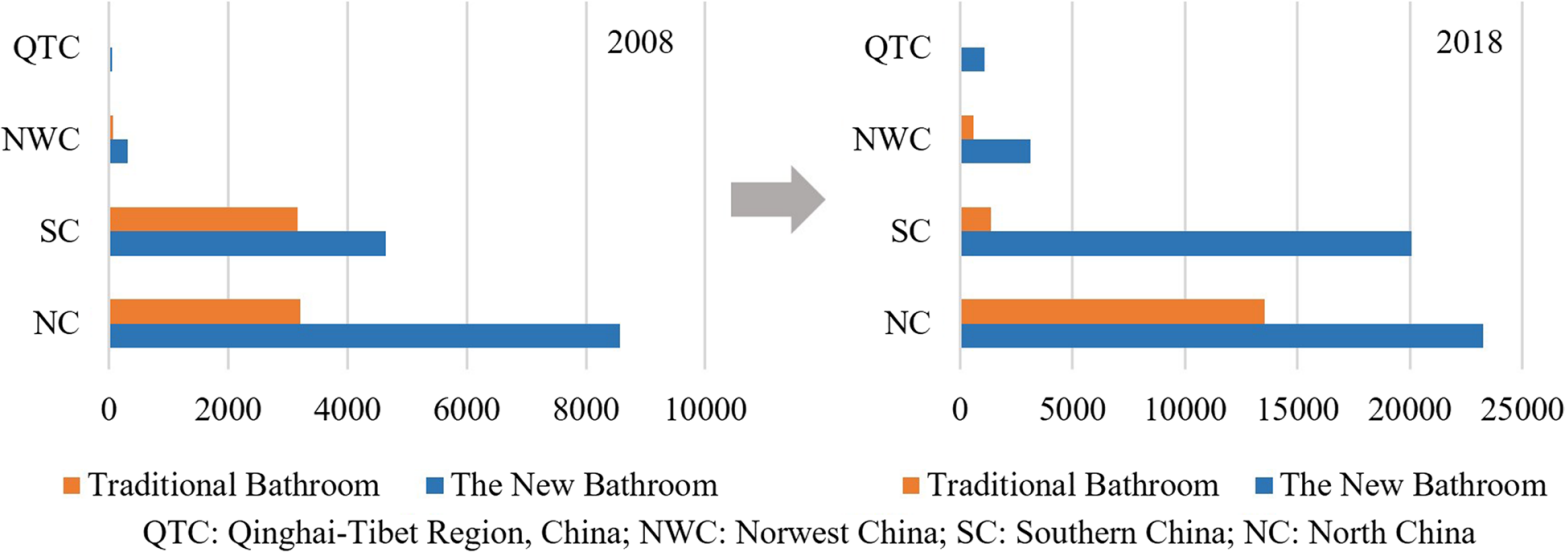
Bath industry and its sub-category growth comparison chart

**Fig. 2:**
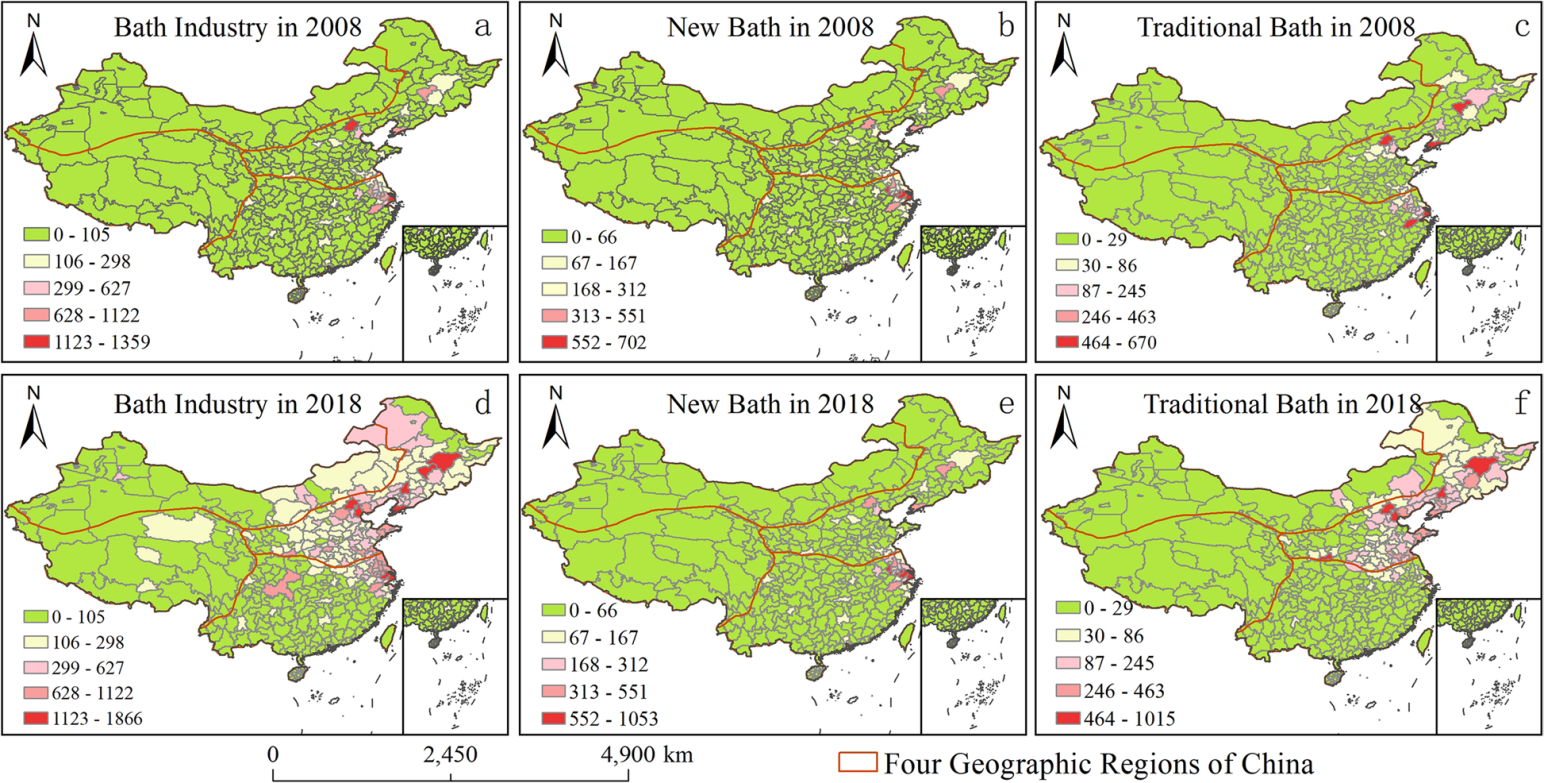
Bath industry and its sub-categories spatial distribution pattern

**Fig. 3:**
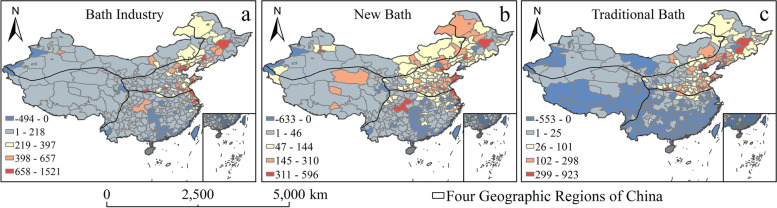
Bath industry and its sub-categories growth chart

The number of bathing places was graded with the help of natural breaks methods. The results showed that the number of bathing places surged, and the proportion of new baths increased. In 2018, there were 63,053 bath places, 2.14 times increase compared with 2008; the number of new bathhouses increased to 47,475, and the proportion increased from 68 to 75%; traditional baths increased to 15,578, and the proportion decreased from 32 to 25% (Fig. 1); secondly, the spatial difference of bathing industry in the four geographical regions was narrowed. The proportion of bathhouses in northern China decreased from 58.7% to 58.3%, and in southern China decreased from 38.9% to 34.0%. While in northwest China, the proportion of bathhouses increased from 2.0% to 5.9% and from 0.4% to 1.8% in the Qinghai-Tibet region (Fig. 1). Third, the spatial pattern of the bath industry has evolved from multi-core to continuous development. In 2008 and 2018, the bath industry exhibited different spatial characteristics of high density in most north and northeast and low density in the rest regions. In high-density areas, new bathhouses form a contiguous development state with Beijing, Tianjin, the Yangtze River Delta, and the capital cities of the eastern provinces as the core (Fig. 2). Finally, the increased range of new bathhouses is more extensive. With more than 47 new bathing growth, the number of cities is 137, including Shenyang, Tianjin, and Chongqing, covering four major geographical areas. There are 96 cities with more than 26 traditional bathing places, covering two major geographic areas in the north and northwest. Traditional bathing places show a significant negative or zero growth in Qinghai-Tibet, the south, and the west of the northwest, including 175 cities (Fig. 3). Because the new bath has bathing, recreation, and health functions, its spatial proliferation is more vital than traditional bathing (Grijota et al., 2021). At the same time, the target customers and market positioning of new baths and traditional baths are different, with the spatial development of new baths mainly in Beijing, Tianjin and Chongqing, while traditional baths mainly in bathing culture output cities in the north, northwest east and south northeast, and restricted in the rest of the region.

#### Spatial dependence pattern

The global Moran's I index for the number of bathing industries from 2008 to 2018 ranged from 0.08 to 0.28, showing an increasing trend; all positive and passing the significance test at the 1% level (Table 2), indicating the increasing spatial aggregation and dependence of the bath industry in China's regions as a whole and the trend of agglomeration in the industrial layout.

**Table 2:** Global Moran's I statistics for bath industry and its sub-categories

Type	Moran's I	*P*-value	Z-score
Baths industry (2008)	0.14	18.87	0.001
Traditional baths(2008)	0.08	12.39	0.001
New baths (2008)	0.18	24.71	0.001
Baths industry (2018)	0.28	36.68	0.001
Traditional baths (2018)	0.21	28.26	0.001
New baths (2018)	0.28	36.39	0.001

**Fig. 4:**
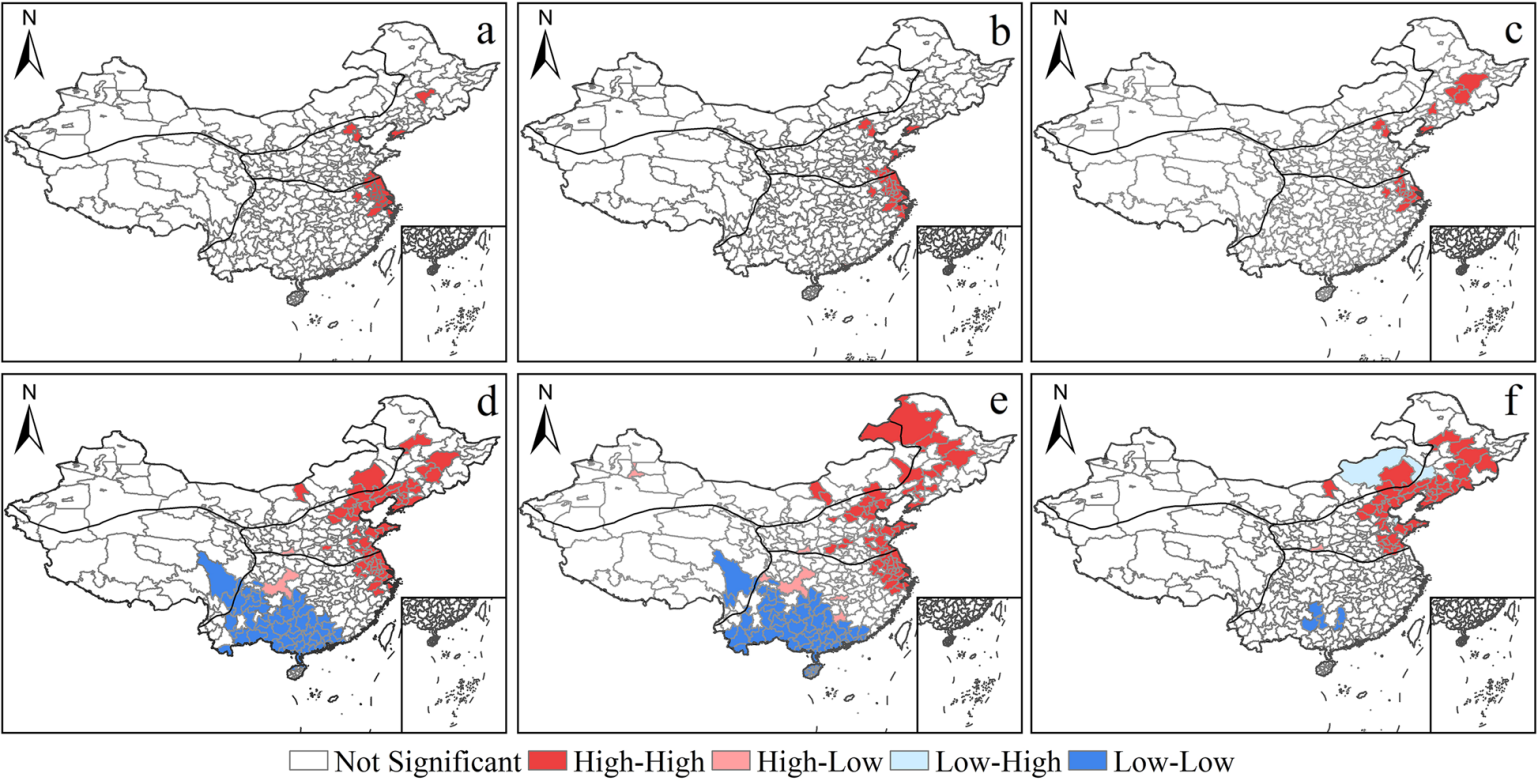
Bath industry and its sub-categories LISA agglomeration map

The number, type, and area of locally significant areas in the bath industry and its sub-categories increased from 2008 to 2018, and the spatial dependence on the local territory increased (Fig. 4). The bath industry is polarized from a small number of significant hotspots to a north–south polarization of significant hotspots and cold spots. The spatial layout of a small number of significant secondary hotspots is transformed. The number of hotspot cities has increased from 22 to 57, forming large, significant hotspot areas such as 'Shenyang Cluster—Beijing-Tianjin-Hebei Cluster' and three isolated hotspots cities as Qiqihar, Baotou, and Zhengzhou in the northern region. Sixty cities with significant cold spots are added in the south, mainly in Sichuan Province, Yunnan Province, and the southern part of Qinghai-Tibet. Xi'an and Chongqing are two secondary cities with significant hot spots (Fig. 4). The new baths are spreading to the north. At the same time, 78 cities are added with significant cold spots, which are similar to the distribution of the bath industry, and five cities with significant secondary hot spots, including Chongqing, Xi'an, and Urumqi, cover bath culture import cities in the south and the west of the northwest (Fig. 4). The traditional bath industry has not formed a large area of significant cold spot areas in the southern region. Compared with the bath industry and the new bath industry, traditional bathing only formed a small significant cold spot agglomeration area in the southern region, including five cities such as Hechi and Liuzhou (Fig. 4). The spatial diffusion of the bath industry mainly takes the bathing culture export city as the radiation center. It spreads to the surrounding cities in the adjacent diffusion mode, forming a state of contiguous development (Xue et al., 2023a, 2023b).

### Influencing factors

#### Impact factor identification and quantification

The development of the bath industry is in line with the classical theory of industrial agglomeration and diffusion (Qi et al., 2021), and the development of the industry is determined by socio-economy and market demand (Li et al., 2019) as well as the diffusion and acceptance of bath culture (Kaltenborn 2020). Therefore, based on these characteristics of the bath industry, this paper explains the influence mechanism of the spatial layout of the bath industry in three dimensions: related industries, market demand, and cultural diffusion (Table 3), with the help of the REF neural network model.

**Table 3:** Index of the impact factor system

Category	Variables	Measurement Index
Affiliated Industries	Restaurant industry spatial correlation(RSC)	Number of catering industry
	Shopping industry spatial correlation(SSC)	Number of shopping industry
	Accommodation industry spatial correlation(ASC)	Number of accommodation industry
	Entertainment industry spatial relevance(ESC)	Number of entertainment industry
Market Demand	Service industry development level(SID)	Tertiary industry output
	City economic development level(CED)	Gross domestic product
	Residents' ability to pay(RAP)	Total retail sales of social consumer goods
	Market volume(MVE)	Population density values
Cultural Communication	Bathing culture input degree(BCI)	Population migration during Spring Festival travel

First, associated industries. The spatial distribution and target customers of the bath industry and the restaurant, accommodation, shopping, and entertainment industries have certain similarities and synergies (Xiang et al., 2022). The bathing industry tends to be located in areas with good development of related industries such as restaurants, accommodation, shopping and entertainment. Therefore, the number of restaurants, lodging, shopping, and entertainment industries in the region is selected to measure the influence level of urban-related industries. The second is market demand.; residents' ability to pay determines the actual and potential market of the bath industry; the population density represents the size of the market (Tang and Ta 2020). Therefore, the urban economic development level, tertiary industry output level, total retail sales of consumer goods, and population density are selected to represent the urban economic development level, service industry development level, residents' ability to pay, and market size. The third is cultural transmission. The spatial migration and flow of the population is the spatial flow of the culture they are loaded with (Repetti & Lawrence, 2021). Bath culture input degree is the amount of people moving into the bathing culture input area during the spring season.

#### Neural network model training results

**Fig. 5:**
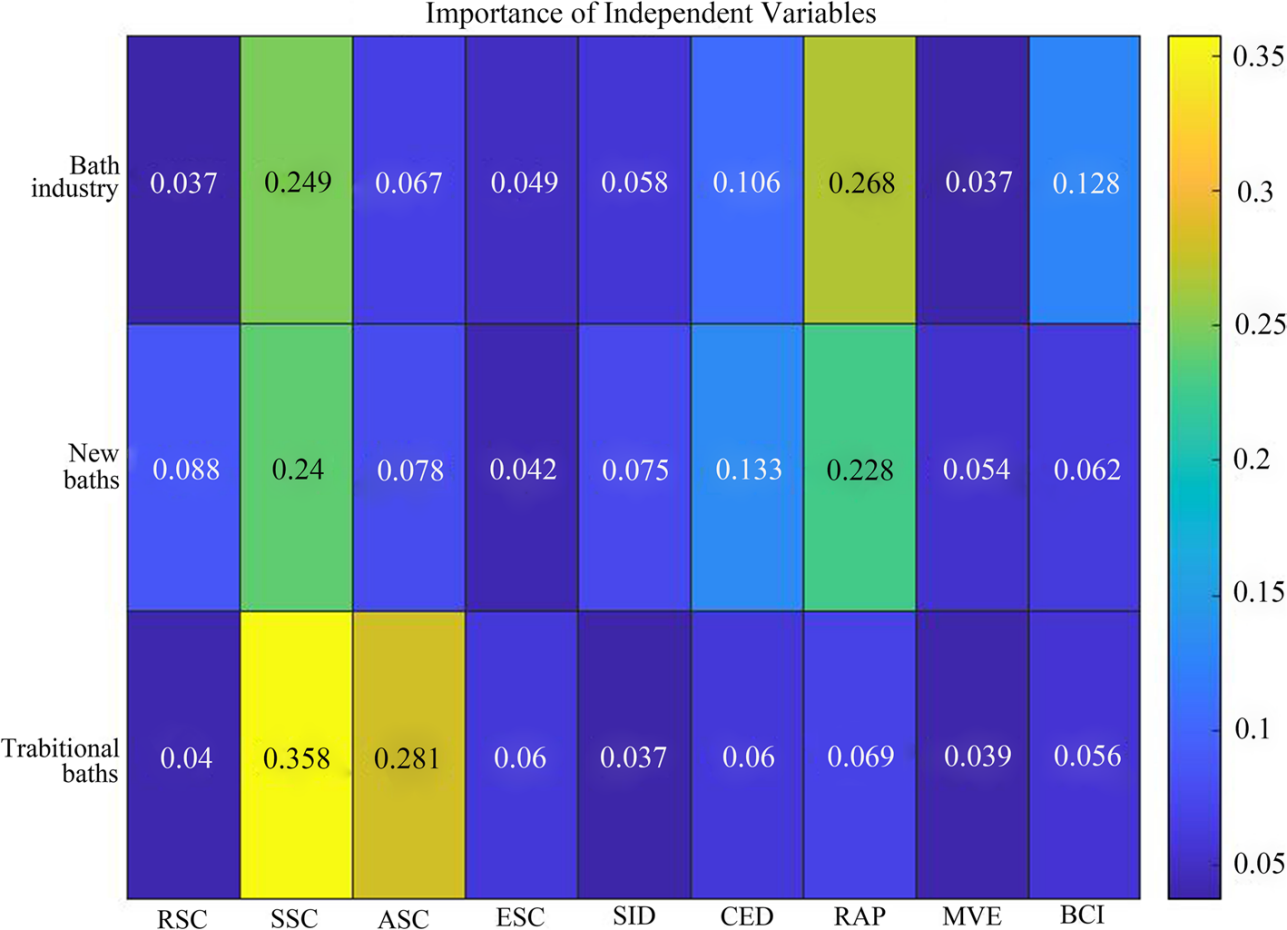
Heat map of the importance of independent variables

The relative errors of the training results for the bath industry and the two sub-categories of new and traditional are 16.5%, 16%, and 17%, respectively, with some reliability. In terms of related industries, the bath industry is most influenced by the catering industry, with an importance coefficient of 0.249, followed by the shopping and entertainment industry (Fig. 5). The bath industry has the lowest association with the accommodation industry, with an importance coefficient of 0.049. Catering, shopping, and entertainment clusters are areas with a high concentration of residential communities and large consumer markets, which can attract bathhouses to locate there (Chen et al., 2021). In addition to being distributed in the economic center, the accommodation industry is also present in large numbers in transportation hubs, tourist attractions or resort areas, etc. The latter is not highly relevant to the bath industry. In terms of market demand, the development of the bath industry is constrained by the economic development of cities and the consumption-ability of residents, with importance coefficients of 0.106 and 0.268, respectively. At the same time, it is less constrained by population density, with an importance coefficient of 0.037. In terms of cultural transmission, the importance coefficient of bath cultural input (0.128) ranks third, indicating that bath cultural input is an essential guiding force for the spatial diffusion of the bath industry.

Regarding industry association, the new bath industry has high synergy with the catering, shopping, and entertainment industries, and the industry chain formed by the three has the possibility of deepening and deepening integration. The poor spatial correlation between the new bath and accommodation industry may be related to the similarity of their service contents. In terms of market demand, the importance coefficients of GDP, retail sales of social consumer goods, and the output value of tertiary industry are 0.228, 0.133, and 0.088, respectively, reflecting that the spatial agglomeration of new baths is strongly constrained by urban economy level and residents' consumption ability, and weakly constrained by population density. Regarding cultural transmission, the importance coefficient of the cultural input degree is 0.062, indicating that the new bathing is influenced by the cultural input degree of bathing to a certain extent.

Compared with new baths, traditional baths have a stronger spatial correlation with the catering and shopping industries and a weaker correlation with the output value of accommodation, entertainment, and tertiary industries, which may since traditional baths mainly provide daily life services. The importance coefficient of bathing culture input degree of traditional bathing is 0.006 lower than that of new bathing, which indicates that the spatial diffusion of traditional bathing is weaker, and the radiation range is smaller, i.e., the traditional bath is strongly dependent on the market of cultural export.

**Fig. 6:**
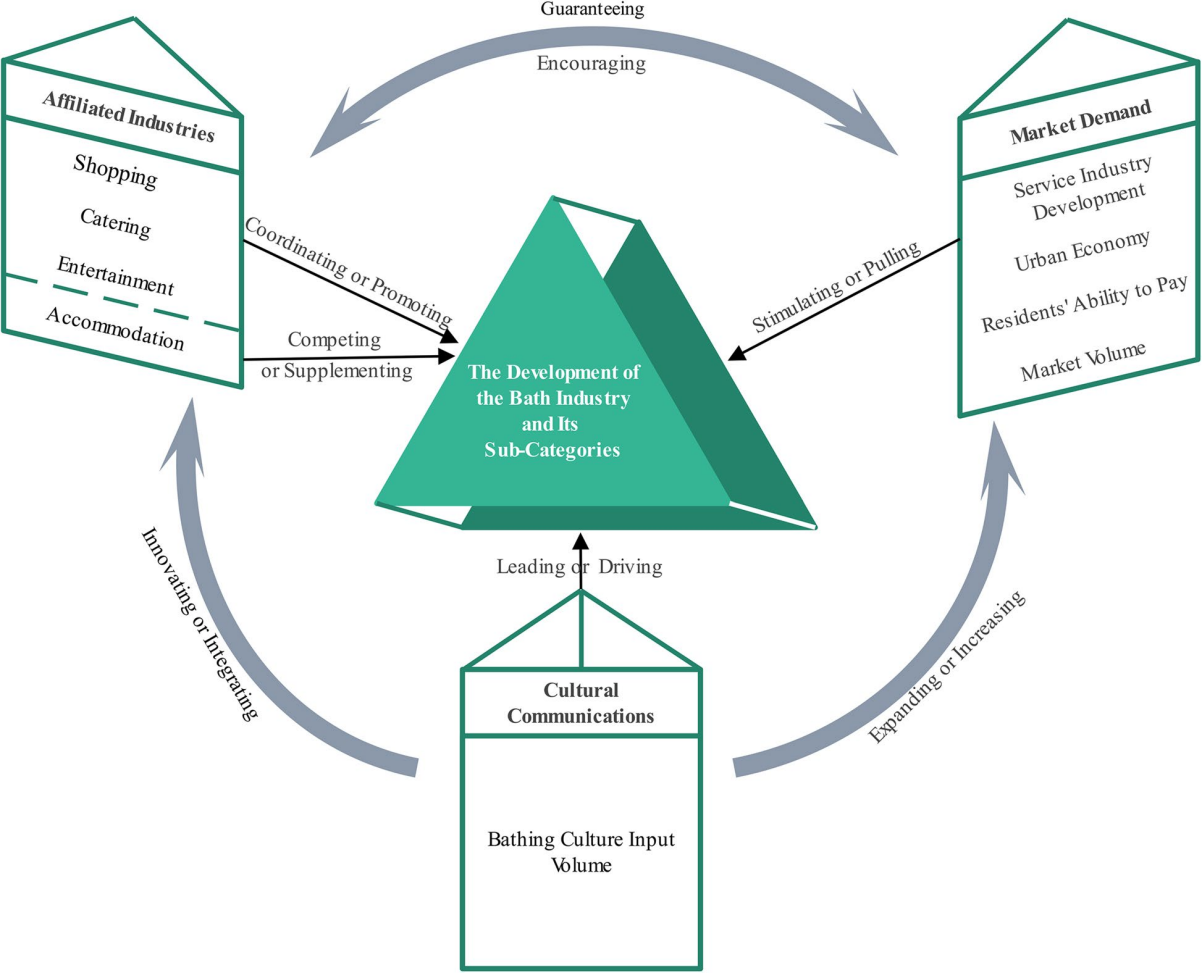
Bath industry development influence mechanism

The influence mechanism of the development of each of the three types of bathing industries is consistent (Fig. 6). The import of bathing culture has a guiding role in the development of the bath industry. Large-scale population flow promotes the exchange and export of various bathing cultures, which leads to the flow of bath industry-related technology and experience, triggering innovation, competition, integration, and service industry development upgrade (Liu et al., 2020). For example, the bath industry and the catering, shopping, and entertainment industries penetrate, cross, and restructure each other and eventually integrate into a new industrial form. However, industries with similar service contents are manifested in market competition or supplementary relationships, such as the accommodation industry. This phenomenon stimulates the development of the urban economy and tertiary industry and enhances the disposable capital and payment ability of residents (Tsai & Chiang, 2019), thus promoting the development of the bath industry. At the same time, market demand and industrial development have a mutually reinforcing relationship. Market demand growth will promote the development, renewal, and upgrading of the bath industry and its related industries. Conversely, the bath industry and its related industries not only improve the city's service industry and health industry, but also promote the development process of the city and promote the market into the "high production and high consumption", which promotes the development of the bath industry and related industries (Xue et al., 2020a, 2020b).

## Discussions

### Applicability of POI data

The accuracy and coverage of data are the basis for measuring the spatial development pattern of the bath industry. The spatial pattern of economic activities is mainly analyzed from field surveys, remote sensing images, and multi-level databases in the traditional data context. For example, Heitz et al. (2018) obtained 207 logistics facilities in Gothenburg, Sweden, by combining data from national institutional databases, field surveys, and satellite remote sensing; Albert et al. (2011) obtained data on manufacturing enterprises in Spain through the Analysis System of Iberian Balances database. The above studies prove the feasibility of using these data for spatial pattern analysis of economic industries, but the problems of data update and coverage still deserve attention. The POI data used in this paper comes from the results of continuous shooting results of GPS-integrated cameras mainly, supplemented by address reverse compilation, enterprise acquisition, and user-spontaneous content, and the information of geographic elements is obtained from multiple channels, and real-time data update (Borromeo & Toyama, 2016). This method effectively improves the accuracy and coverage of the data. Therefore, it is feasible and credible to measure the spatial development pattern of the bath industry with the help of POI data. The combination of multi-source data is an effective way to excavate the development law of the bath industry deeply. 'The Ministry of Commerce's Guidance on the Standardized Development of Bath industry' mentions the need to further improve the service quality and safety and hygiene level of the bath industry; POI data only contain basic information such as the names and geographical locations of bathhouses, which cannot be classified and evaluated according to the service quality and safety and hygiene level. For example, Wu et al., (2021a, 2021b) combined POI data and global land cover products to identify the core drivers of urban land use change; Wang et al. (2016) used POI data to respond to the distribution of urban facilities and social media data to respond to the topics discussed in different regions, and the two were combined to infer urban land use patterns. The next step of this paper will analyze the spatial pattern of service quality and safety and hygiene level in the bath industry by using the comprehensive rating value of bathhouses and the safety and hygiene rating value of public reviews to represent service quality and safety and hygiene level.

### Comparison of influence factor systems and measurement methods

Establishing a comprehensive and measurable system of influencing factors is the key to analyzing the spatial development pattern of the industry. The change of industrial spatial pattern is the function of a compound influence mechanism; for example, Rubalcaba et al. (2013) selected the factors influencing the location of commercial services in major European cities from the dimensions of urban economic development, market demand, and city image; Zhu (2021) showed that cultural communication has a specific role in promoting the spatial development of local snacks. This paper further quantifies the influence factor of the "cultural communication degree" with the help of Tencent location big data to precisely measure its strength on the spatial development of the bath industry and further quantifies the influence factor of 'cultural communication degree' and precisely measures its promotion strength to the spatial development of the bath industry. The 'Health China 2030' plan mentioned that the government should take the lead and form an institutional system, so it is also essential to observe and quantify the degree and effect of government intervention to improve the influence factor system. The continuous optimisation of the methodology is a guarantee for improving the accuracy of the measurement results. Taking the bathing industry as an example, this paper finds that neural networks cannot account for the magnitude and direction of the influencing factors and cannot further explain the reasons for the formation of the industry's spatial development pattern. However, the neural network has strong computational power and is suitable for large volume data analysis (Anneleen et al., 2017; Zhang et al., 2023). In the context of big data, combining deep learning methods such as neural networks and regression models, etc., can deeply analyze the causes of the formation of geographical phenomena and discover more geographical knowledge (Lin & Billa, 2021; Pei et al., 2021).

### Policy implications

It is found that the bath industry and its subcategories have a high spatial development level in Northern and eastern Northwest China, while the rest areas need to be developed. It also shows that the new bath industry has a more comprehensive spatial development range and greater intensity than the traditional bath industry. Based on the above findings, this paper proposes the following policy recommendations. First, Improve the adaptability and integration of the bath industry; Gilboa and Jaffe (2021) suggest that one product is not suitable for all people, and the diversity and adaptability of products should be improved by combining the preferences and needs of different residents. Therefore, the bath industry can be integrated with various cultures, such as pedicure, foot massage, steam, and mud bath, to increase the popularity and acceptability of the industry in the local area. Second, traditional baths can expand the market by improving service quality (Tang et al., 2021). Traditional baths need to find their market positioning, increase various service items, improve service quality and hygiene level, change marketing strategies to improve customer flow and customer return rate, and achieve scale development and standardized operation under the 'Guidance of the Ministry of Commerce on the Standardized Development of Bath industry'. Third, bathhouses should improve the service system and risk management control in the epidemic context. COVID-19 has brought a sizeable economic impact on the bath industry. In the case of a controlled epidemic, bathhouses can advocate scenario-based and cultural consumption to increase customer flow based on good epidemic prevention and control. Bathhouses need excellent risk management control to avoid economic losses caused by irresistible factors.

## Conclusion

This study identifies the evolution of the spatial pattern of the bath industry at two scales: four geographic regions and prefecture-level cities based on multi-source data such as POI, population migration, and socio-economic statistics. Moreover, this paper constructs a system of influencing factors from three dimensions: related industries, market demand, and cultural communication, and explains the effectiveness of the influences with the help of REF neural network models. The following conclusions are drawn: first, based on the case study of the development of the bath industry and its sub-categories in mainland China, this paper finds that the integrated application of multi-source data has a more tremendous potential to study the spatial pattern and influencing factors of the industry at multiple scales, especially in the acquisition and analysis of spatial data of the local characteristic industries; second, the bath industry and its subcategories have a high spatial development level in Northern and eastern Northwest China, while the rest areas need to be developed.. It also shows that the new bath industry has a more comprehensive spatial development range and greater intensity than the traditional bath industry, and third, to achieve the goals of developing the health industry in the policy documents such as 'Health China 2030' and 'Guidance of the Ministry of Commerce on Standardizing the Development of Bath industry', we should promote the cross-fertilization of bathing culture and various local cultures, to improve the service level of bath industry, expand the consumer market and realize the healthy and balanced development of bath industry.
